# Presence of Viral RNA and Proteins in Exosomes from Cellular Clones Resistant to Rift Valley Fever Virus Infection

**DOI:** 10.3389/fmicb.2016.00139

**Published:** 2016-02-11

**Authors:** Noor A. Ahsan, Gavin C. Sampey, Ben Lepene, Yao Akpamagbo, Robert A. Barclay, Sergey Iordanskiy, Ramin M. Hakami, Fatah Kashanchi

**Affiliations:** ^1^National Center for Biodefense and Infectious Diseases, School of Systems Biology, George Mason University, ManassasVA, USA; ^2^Ceres Nanosciences, Inc., ManassasVA, USA; ^3^Laboratory of Molecular Virology, George Mason University, ManassasVA, USA

**Keywords:** Rift Valley Fever Virus, exosomes, resistant clones

## Abstract

Rift Valley Fever Virus (RVFV) is a RNA virus that belongs to the genus *Phlebovirus*, family *Bunyaviridae*. It infects humans and livestock and causes Rift Valley fever. RVFV is considered an agricultural pathogen by the USDA, as it can cause up to 100% abortion in cattle and extensive death of newborns. In addition, it is designated as Category A pathogen by the CDC and the NIAID. In some human cases of RVFV infection, the virus causes fever, ocular damage, liver damage, hemorrhagic fever, and death. There are currently limited options for vaccine candidates, which include the MP-12 and clone 13 versions of RVFV. Viral infections often deregulate multiple cellular pathways that contribute to replication and host pathology. We have previously shown that latent human immunodeficiency virus-1 (HIV-1) and human T-cell lymphotropic virus-1 (HTLV-1) infected cells secrete exosomes that contain short viral RNAs, limited number of genomic RNAs, and viral proteins. These exosomes largely target neighboring cells and activate the NF-κB pathway, leading to cell proliferation, and overall better viral replication. In this manuscript, we studied the effects of exosome formation from RVFV infected cells and their function on recipient cells. We initially infected cells, isolated resistant clones, and further purified using dilution cloning. We then characterized these cells as resistant to new RVFV infection, but sensitive to other viral infections, including Venezuelan Equine Encephalitis Virus (VEEV). These clones contained normal markers (i.e., CD63) for exosomes and were able to activate the TLR pathway in recipient reporter cells. Interestingly, the exosome rich preparations, much like their host cell, contained viral RNA (L, M, and S genome). The RNAs were detected using qRT-PCR in both parental and exosomal preparations as well as in CD63 immunoprecipitates. Viral proteins such as N and a modified form of NSs were present in some of these exosomes. Finally, treatment of recipient cells (T-cells and monocytic cells) showed drastic rate of apoptosis through PARP cleavage and caspase 3 activation from some but not all exosome enriched preparations. Collectively, these data suggest that exosomes from RVFV infected cells alter the dynamics of the immune cells and may contribute to pathology of the viral infection.

## Introduction

Rift Valley Fever Virus (RVFV; *Bunyaviridae: Phlebovirus*) is an enveloped virus that contains three single stranded RNA segments. The virion is approximately 80–120 nm in diameter (Ikegami, 2012) with a lipid bilayer envelope containing glycoproteins Gn and Gc. The RVFV genome is tripartite and consists of L, M, and S segments. The L and M segments, respectively, encode for the RNA dependent RNA polymerase (L protein) and the NSm and 78-kDa proteins and two major envelope proteins, Gn and Gc. The S segment is of ambisense polarity and encodes for two proteins: nucleoprotein (N) and a non-structural protein (NSs; Pepin et al., 2010). The three ribonucleocapsids (RNPs) are filamentous and exhibit pan-handle like structure owing to complementary genome terminal sequences. This acts as a promoter for genomic RNA synthesis since they lack a 5′ cap structure. Transcription of each of the segments utilizes a cap-snatching process by cleaving the 5′cap from the host mRNA (Ikegami, 2012).

The critical viral protein NSs has been shown to form filaments in the nuclei of infected cells (Yadani et al., 1999). NSs interacts with the p44 subunit of a transcription factor (TFIIH) responsible for transcription of cellular RNA polymerase I and II. This interaction most likely shuts down the assembly of the TFIIH complex, therefore resulting in general transcriptional shutoff (Le May et al., 2004). RVFV infection is known to activate certain transcription factors (IRF-3, AP-1, and NF-κB); however, there has also been evidence of suppression of specific genes including the IFN-β and IFN-related genes (Billecocq et al., 2004). NSs specifically interacts with Sin3A-associated protein (SAP30) and forms a multiprotein complex on the IFN-β promoter, resulting in overall suppression (Le May et al., 2008). In addition, NSs has the capability to degrade host proteins such as the double stranded RNA-dependent protein kinase (PKR; Habjan et al., 2009) and TFIIH p62 (Kalveram et al., 2011).

Exosomes are cell derived membrane bound vesicles secreted into the surrounding extracellular environment. Exosomes were originally considered as a vehicle to remove cellular waste from the cytosol; however, in recent years they have been recognized to also aid in cell–cell communication, play a critical role in immunomodulation, and enhance infectivity during viral infections (Colino and Snapper, 2007; Izquierdo-Useros et al., 2010; Luga et al., 2012; Fleming et al., 2014). Exosomes are characterized as extracellular vesicles ranging from 50 to 150 nm in diameter and a density of 1.23–1.16 g mL^-1^ (Théry et al., 2006). They are thought to originate from late endosomes containing specific mRNAs, microRNAs, proteins, and lipids that are then secreted into the extracellular environment (Harding et al., 1984; Jaworski et al., 2014a; Ailawadi et al., 2015; Izumi et al., 2015). The exact content of the exosomes vary depending on the cellular origin; however, some of the more common exosomal markers include CD63, Alix, and actin (Logozzi et al., 2009).

In recent years, the role of exosomes from virally infected cells has been explored with great interest. For instance, virally infected cells produce exosomes with major changes in protein composition as compared to exosomes from uninfected cells (Meckes et al., 2013). In particular, this is demonstrated in exosomes purified from B cells infected with Kaposi sarcoma herpesvirus (KSHV), Epstein–Barr Virus (EBV), or cells that are dually infected. Mass spectrometry results indicated that 871 proteins were identified and approximately 360 proteins were unique to exosomes isolated from virally infected B cells. It is believed that viral latent membrane protein 1 (LMP1) upregulates the protein content in exosomes from EBV infected cells. These exosomes can affect the interferon and NF-κB signaling pathways, lipid raft organization, and trafficking of proteins. On the other hand, the effects of exosomes from KSHV infected cells differed in terms of protein translation, metabolism, and cell migration. Additionally, exosomes released from virally infected cells have been shown to have varying compositions of RNA (Canitano et al., 2013). For instance, exosomes released from EBV infected lymphoblastoid and nasopharyngeal cells contain EBV encoded latent phase mRNAs.

RNA viruses such as retroviruses can hijack exosomal proteins as well as other components to increase their spread throughout the body. In addition to this benefit, the resulting changes in recipient cells that receive the exosomes can be profound, leading to disease state and pathologies associated with an increased infection. To this end, we have previously shown that latent human immunodeficiency virus-1 (HIV-1) and human T-cell lymphotropic virus-1 (HTLV-1) infected cells produce exosomes with remarkable phenotypes important in cellular activation and viral spread. For instance, HIV-1infected latent cells secrete exosomes containing TAR RNA, a 52-base stem-and-loop structure transcribed from the integrated provirus. TAR RNA was found to be associated with components of the miRNA machinery that are involved in generation of specific 5′ and 3′ miRNAs, as well as in sera of patients on antiretroviral drugs and long-term non-progressors (LNTPs; Narayanan et al., 2013). The net effects of these RNAs is to act as a decoy in the recipient cells to suppresses innate immune molecules such as PKR, and activate NF-κB through TLR pathways (Sampey et al., 2015). A similar scenario is also seen with HTLV-1 infected latent cells where Tax protein, a powerful transactivator of NF-κB and CREB factors, stimulates recipient cells, potentially leading to activation of pathways needed for transformation (Jaworski et al., 2014a). Additionally, HTLV-1 exosomes contain high levels of cellular miRNAs that are bound to Ago2, but have limited amounts of Dicer and Drosha (unpublished data). The increased level of Ago2 bound to cellular miRNAs suggests that HTLV-1 infected exosomes can rapidly control mRNA and their subsequent inhibition in the recipient cells.

In the current manuscript, we asked whether cell clones resistant to cytotoxic effects of RVFV infection secreted exosomes that could potentially regulate neighboring cells. Similar to HIV-1 and HTLV-1 latently infected cells, we generated latent resistant clones *in vitro* following RVFV infection by isolating clones that survived the infection. These resistant clones were also resistant to subsequent RVFV infection, but not to other viruses, including VEEV. The exosomes from these clones were enriched and characterized for presence of viral genomic RNA and proteins. We found that almost all exosome-enriched secretions from resistant clones contain viral RNA and proteins such as N- and NSs. The net effect of exosome-enriched preparations that contained both viral RNA and protein were induction of apoptosis in recipient cells including T- and monocytic cells. The mechanism of apoptosis was related to regulators such as PARP and caspase 3. The implication of how exosomes may be controlling neighboring cells and their contribution to pathogenesis of RVFV will be discussed.

## Materials and Methods

### Cell Culture and Reagents

Vero (African green monkey kidney) cells were maintained in Dulbecco’s modified minimum essential medium (DMEM) supplemented with 10% fetal bovine serum (FBS), 1% penicillin/streptomycin, and 1% L-glutamine. Exosome free DMEM was supplemented as above except FBS was ultra-centrifuged at 100,000 × *g* for 70 min to remove bovine exosomes. To generate resistant clones, Vero cells were infected with RVFV at a multiplicity of infection (MOI) of 3. Following a 2 weeks culture (with addition of media and presence of virus in the supernatant), the individual colonies (∼1% of cells) resistant to infection were selected. They were isolated using sterile pipette tips and trypsin. Clones were plated and passaged 50 times to further purify individual clones. The assay was repeated twice, once with the wild type MP12 and then repeated independently with V5- and Flag-tagged-MP12 virus. In this manner, two sets of resistant clones were isolated either containing wild type MP-12 or V5- and Flag-tagged MP-12 resistant clones.

The Jurkat T cell line was isolated from an adolescent male patient with acute T cell leukemia (Schneider et al., 1977), while the CEM T cell line was isolated from a juvenile female presenting acute lymphoblastic leukemia (Foley et al., 1965). Both of these T cell lines carry mutations within the p53 gene (Laumann et al., 1992; Park et al., 1994; Cinti et al., 2000; Ahmadianpour et al., 2013). The U937 monocytic cell line was derived from an adult male patient with histiocytic lymphoma (Sundstrom and Nilsson, 1976). As with the Jurkat and CEM cell lines, the U937 cell line also harbors mutations within the p53 gene (Sugimoto et al., 1992; Mori et al., 1997).

### Isolation of Exosomes

Resistant clones were expanded into two T-150 flasks and incubated at 37°C for 5 days. One hundred milliliter of exosome free DMEM was used for growth of cells. Supernatants were centrifuged at 2,000 rpm for 10 min at 4°C to eliminate dead cells. Supernatants were then filtered through 0.22 μm filters to remove most apoptotic bodies, but allow viruses and exosomes to pass through the filter. The filtrate was then processed through a series of ultracentrifugation steps. In the first step, filtrate was ultracentrifuged at 10,000 × *g* for 30 min at 4°C. Supernatants were transferred to clean ultracentrifuge tubes and ultracentrifuged again at 100,000 × *g* for 70 min at 4°C. Supernatants were removed and exosome pellets were resuspended in PBS without calcium and magnesium and ultracentrifuged again at 100,000 × g for 70 min at 4°C. Pellets were resuspended in 50–100 μl of sterile PBS without calcium and magnesium. These semi-purified exosomes were stored at 4°C for up to 2 weeks for subsequent analysis. The protein concentrations of exosome preparations were determined by running Bradford assays on exosomal lysates.

For exosome isolations using low volumes, we utilized nanoparticles. Nanotrap particles NT080 and NT082 (Ceres Nanosciences) were used in combination to enrich for exosomes. Equal amounts of nanoparticles were mixed together (∼0.5 ml each) and resuspended in a 30% slurry in PBS without Calcium and Magnesium. Twenty microliters of the slurry was added to 100–1000 μl of supernatant and rotated either overnight at 4°C or for 30 min at room temperature. Samples were centrifuged at 14,000 rpm for 5 min. Supernatants were aspirated and washed twice with PBS, and finally resuspended in 30 μl prior to subsequent assays. The samples were used for RNA extraction using the trizol-chloroform method or for western blots.

Dynabeads coated with CD63 antibody (Life Technologies) were used to purify exosomes from cell supernatant. Twenty five microliters of the suspension of magnetic dynabeads were washed with PBS and used for each sample. Approximately 1 ml of 5 day-culture supernatants were added to the beads and incubated overnight at 4°C. The bead-bound exosomes were washed twice with PBS and subsequently used for RNA isolation.

### RT-PCR

Total RNA was extracted from different samples including exosomes and whole cell lysates via trizol-chloroform method. Approximately 400 ng/μl of RNA was used from sample for cDNA synthesis with GoScript Reverse Transcription System (Promega) using Random Primers. Primer pairs utilized for each segment were as follows: for M segment: Gn Forward (5′-AAA GGA ACA ATG GAC TCT GGT CA-3′, *T*m = 58°C) and Gn Reverse (5′-CAC TTC TTA CTA CCA TGT CCT CCA AT-3′; *T*m = 58°C); for L segment: RVFV L Polymerase Forward (5′-GGT GGC ATG TTC AAT CCT TT-3′; *T*m = 54°C) and RVFV L Polymerase Reverse (5′-GCA TTC TGG GAA GTT CTG GA-3′; *T*m = 54°C); for S segment: RVFV NSs Forward (5′-TCT GAA AGA AGC CAT ATC CT-3′; *T*m = 54°C) and RVFV NSs Reverse (5′-CTC GCT ATC ATC CTG TGT AA-3′; *T*m = 54°C) and RVFV N Forward (5′-CAT GGT GGA TCC TTC TCT AC-3′; *T*m = 54°C) and RVFV N Reverse (5′-CTA TTC ACT GCT GCA TTC AT-3′; *T*m = 54°C). The PCR conditions were: one cycle at 95°C for 2 min, 41 cycles at 95°C for 15 s and 58°C or 54°C (depending on primer set) for 40 s. The absolute quantification of the samples was determined based on the cycle threshold (Ct) value relative to the standard curve. The dilutions of plasmids containing sequences of the segments of RVFV genome cDNA were used as quantitative standards.

### Western Blot Analysis

For analysis of protein content of exosomes released from RVFV resistant clones, exosomes were isolated via the differential centrifugation method. Four microgram/sample were added to SDS sample buffer supplemented with 10% 2-mercapthoethanol. Entire content of sample was loaded onto a 4–20% Tris-Glycine gel run at 150 V, followed by overnight transfer onto PVDF membranes. Membranes were blocked in 5% milk in PBS plus 0.1% tween-20 for 2 h at room temperature, then incubated overnight at 4°C with appropriate antibody: α-CD63 (ab8219 1:500), α-N protein (generous gift or Dr. C. Schmaljohn; USAMRIID), α-Flag (sc-807 1:200), α-Alix (sc-49268 1:150), and α-beta actin (ab49900 1:5000).

### TLR3 Assay

The HEK-293T based reporter cell line, HEK-Blue hTLR3 (InvivoGen), was used to detect activation of TLR3 by supernatant of resistant clones. HEK-Blue hTLR3 cells contain a secreted embryonic alkaline phosphatase (SEAP) reporter gene that is transcribed upon TLR3 activation. Prior to adding the HEK-Blue hTLR3 cells, experimental samples and controls were brought up to 20 μL using PBS and distributed in a 96-well plate. HEK-Blue hTLR3 cells were grown to 50–80% confluence in a T-75 flask, washed with PBS, and then detached with 2 mL of pre-warmed PBS and tapping the flask. Cells were counted and then resuspended to a cell density of 2.8 × 10^5^ cells/mL in pre-warmed HEK-Blue Detection medium (InvivoGen). Next, 180 μL of the resuspended cells were added to each of the samples in the 96-well plate to give a final cell count of 5 × 10^4^ cells/well. After 18–68 h of incubation (depending on the experimental design) at 37°C in 5% CO_2_, the absorbance (600 nm) of each control and test condition in the 96-well plate was measured using the GloMax Multi Detection System (Promega). Readings from all positive controls and experimental samples were normalized using the mean reading from three PBS negative controls.

### Cell Viability Assay

Approximately 50,000–100,000 cells in 50 μl exosome free media were plated in a 96 well plate. Fifty microliters of supernatant from resistant clones were centrifuged 10 min at 14,000 rpm, added to plated cells and allowed to incubate at 37°C in 5% CO_2_ for 5 days. Plate and Cell Titer Glo reagent (Promega) were allowed to cool/thaw at room temperature for 30 min. One hundred microliters of Cell Titer Glo reagent was added to appropriate wells and manually shaken for 2 min, followed by a 10 min incubation at room temperature. Counts from the exosome free DMEM served as background and were used to normalize values. Assays were conducted in biological triplicates. Approximately 50,000–100,000 cells (Jurkat or U937) in 50 μl exosome free RPMI-1640 medium were plated in a 96 well plate. Exosomes isolated through ultracentrifugation were added at 2 μg/well. Plates were incubated at 37°C for 5 days. One hundred microliters of Cell Titer Glo reagent were added to appropriate wells and manually shaken for 2 min, followed by incubation at room temperature for 10 min.

### Statistical Analysis

Standard deviation was calculated in all quantitative experiments. *P*-values were calculated by the student’s *t*-test and considered to be statistically significant when *p* < 0.05 unless otherwise noted.

## Results

### Generation of RVFV Resistant Clones

**FIGURE 1:**
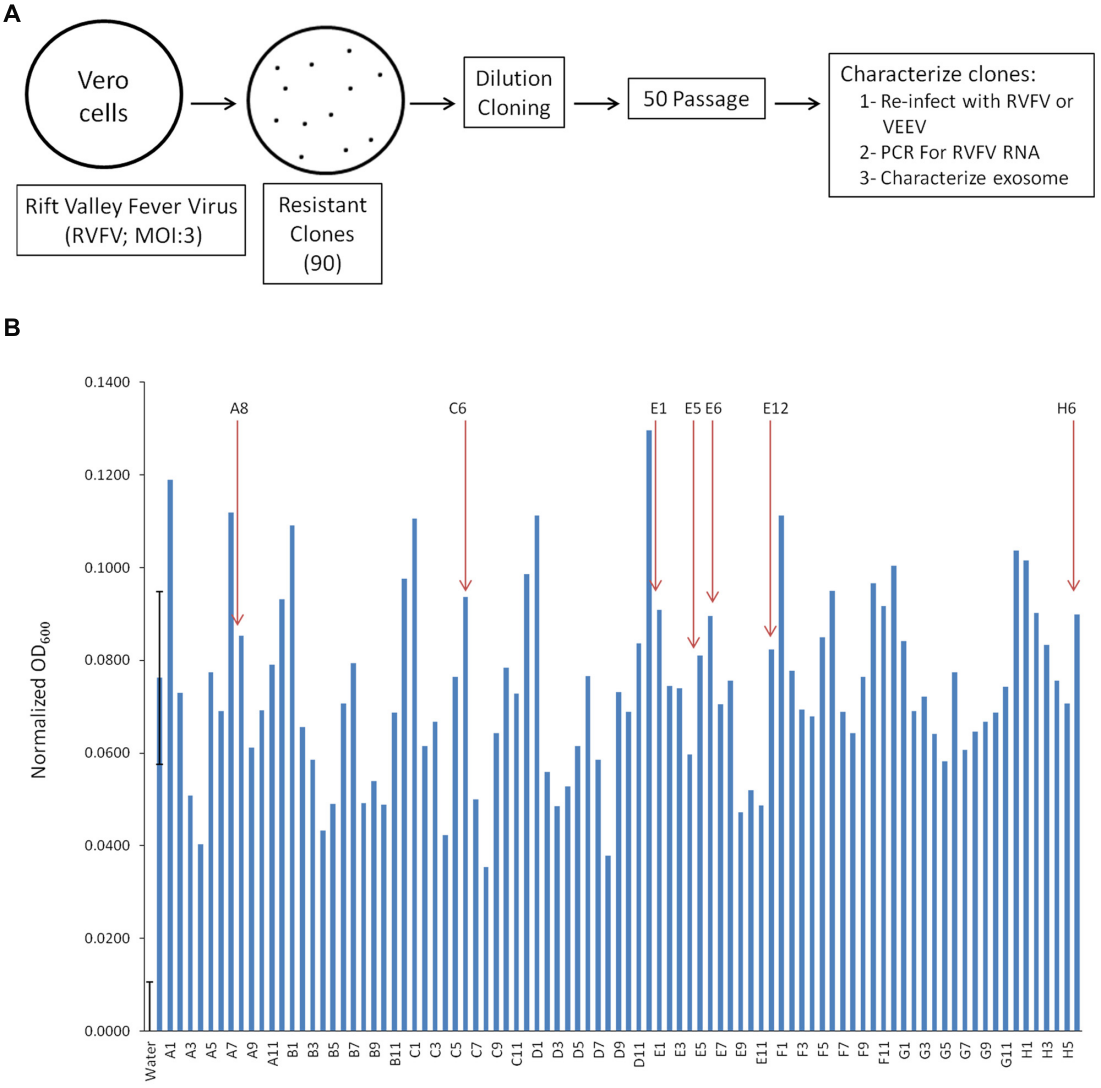
**Generation of Rift infected resistant clones.** **(A)** Vero cells were infected with RVFV at MOI 3. Approximately 1% of cells survived the infection and isolated using trypsin diluted with PBS. Clones were plated and passaged 50 times before characterization. **(B)** The HEK-293T based reporter cell line, HEK-Blue hTLR3 (InvivoGen), was used to detect activation of TLR3 by supernatant of RVFV resistant Vero cells. After 18 h of incubation at 37°C in 5% CO_2_, the absorbance (600 nm) of each control and test condition in the 96-well plate was measured using the GloMax Multi Detection System (Promega). Readings from all positive controls (sample 2, 10 ng/mL Poly I/C) and experimental samples were normalized using the mean reading from three sterile water treated negative controls. Seven clones (as indicated) were selected for follow up experiments. Error bars on the first two samples (negative and positive controls) indicated ±1 SD of biological triplicates.

Exosomes are known entities that are involved in communication between cells resulting in spread of information, including cytokines and miRNA, some of which aid in regulating infection of adjacent cell (Fleming et al., 2014; Kalamvoki et al., 2014). We were interested in characterizing exosome-enriched preparations from RVFV resistant clones that were incapable of releasing functional virions and asked whether they could influence the neighboring cells. We decided to generate resistant clones since RVFV infection causes cell death in a majority of infected cell types *in vitro* within 24–48 h. Therefore, we devised a scheme to generate reliable clones that contained viral RNA and/or proteins that could potentially be secreted from the cells as components of extracellular vesicles, particularly exosomes. Our rationale comes from our previous work where HIV-1 or HTLV-1 infected resistant clones (i.e., latent cells) produced exosomes that contained both viral RNA and/or proteins (Narayanan et al., 2013; Jaworski et al., 2014a). Specifically, we infected Vero cells with RVFV at MOI of 3 and allowed the cells to incubate for 10–14 days at 37°C days and checked for presence of small surviving resistant clones. These cultures contained individual patches of resistant cells despite having high virus titer in the media. Following 2 weeks incubation, plates were washed and cells were removed using a micropipette with a solution of trypsin and diluted in a microtiter plate to achieve a 1–10 cell/well cultures (**Figure 1A**). We created 90 resistant clones using this dilution cloning procedure. These cells were next expanded and supernatants (5 days incubation at 37°C) were added to TLR3 indicator cells. We and others have previously shown that molecules within the exosomes activate various cascades of signal transduction as evident by increase in pro-inflammatory cytokines mediated by TLRs (Bhatnagar and Schorey, 2007; O’Neill and Quah, 2008). We focused on seven of 90 clones that were amongst the top 30 activators of the TLR3 reporter cell line and survived 50 subsequent passages to increase their clonal purity (**Figure 1B**). To further assure that the passaged clones were truly resistant to infection, they were re-infected with RVFV (MOI: 3). After 48–72 h, none of these clones demonstrated signs of cell death following secondary viral infection. However, all of the clones were sensitive to secondary infection by another cytoplasmic RNA virus, the Venezuelan Equine Encephalitis Virus (data not shown). We finally performed a set of confirmatory assays including RNA PCR to validate presence of RVFV genome in these clones (see below). Collectively, these data imply that RVFV infection can allow generation of cells that are resistant to RVFV infection.

### Presence of RVFV Genome in Resistant Clones

**FIGURE 2:**
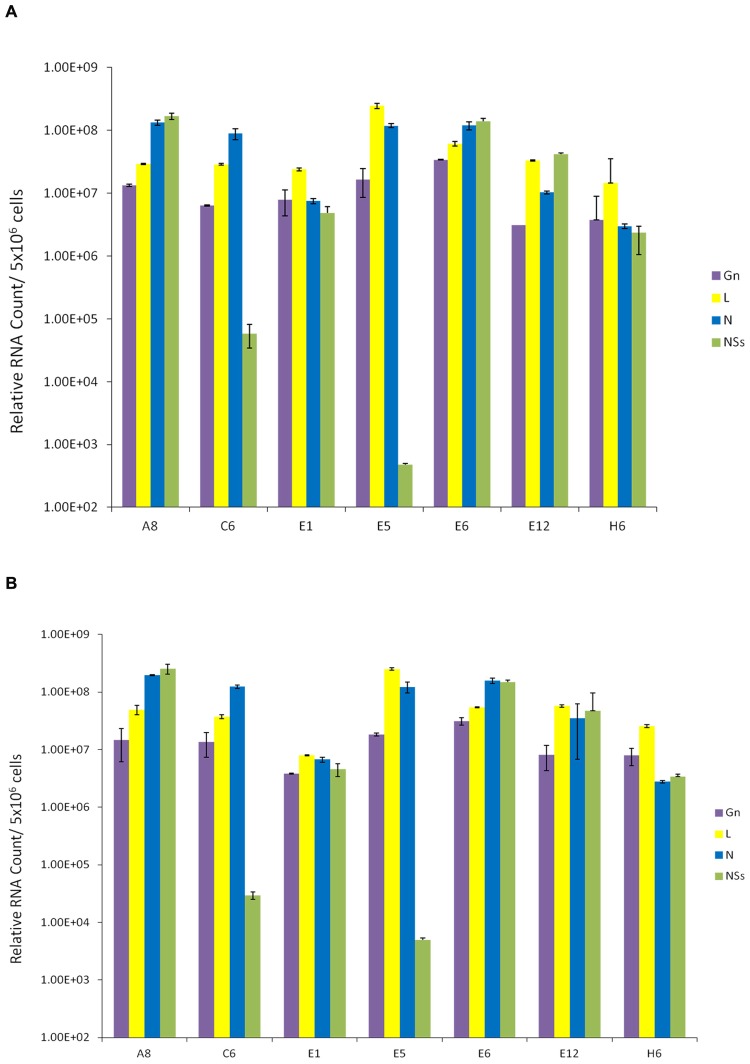
**Presence of RVFV genome in resistant clones.** **(A)** Total RNA was extracted from seven RVFV resistant Vero cells using the trizol-chloroform method. For each sample, approximately 400 ng/μl of RNA was used for cDNA synthesis with GoScript Reverse Transcription System using Random Primers. The absolute quantification of the samples was determined based on the cycle threshold (Ct) value relative to the standard curve. **(B)** qRT-PCR analysis of resistant clones 10 days after secondary infection with RVFV at MOI 1.

We next asked whether these resistant clones contained RVFV genome within the cells. We also asked whether viral genome levels were altered following a second RVFV infection of these cells. Our rationale for these experiments was that if these clones were resistant to a new incoming virus replication, the overall dynamics of the intracellular RVFV genomes in these clones should not change drastically. Total RNA was extracted from the clones and cDNA was generated by RT reaction and then quantitated by real-time PCR. A qRT-PCR analysis of cellular RNA from clones prior to and after infection was performed using primer pairs for each of the three viral segments. The primers specific for the polymerase gene detected L segment; Gn gene-specific primer set detected the M segment; and, the S segment was detected using the primers for both N and NSs genes. Results of qRT-PCR indicated that all clones contained intracellular M, L, and S segments of viral genomic RNA; however, varying levels of the NSs RNA were present in few of the clones (**Figure 2A**). More importantly, when these cells were incubated with RVFV again and carried for 10 days, there were no dramatic changes in the genome content of the resistant clones (**Figure 2B**), indicating that these clones maintain viral genome integrity without much alteration after a new round of cell duplication or infection. Collectively, these results imply that the resistant clones carry RVFV genome and the cells are resistant to apoptosis and re-infection by RVFV.

**FIGURE 3:**
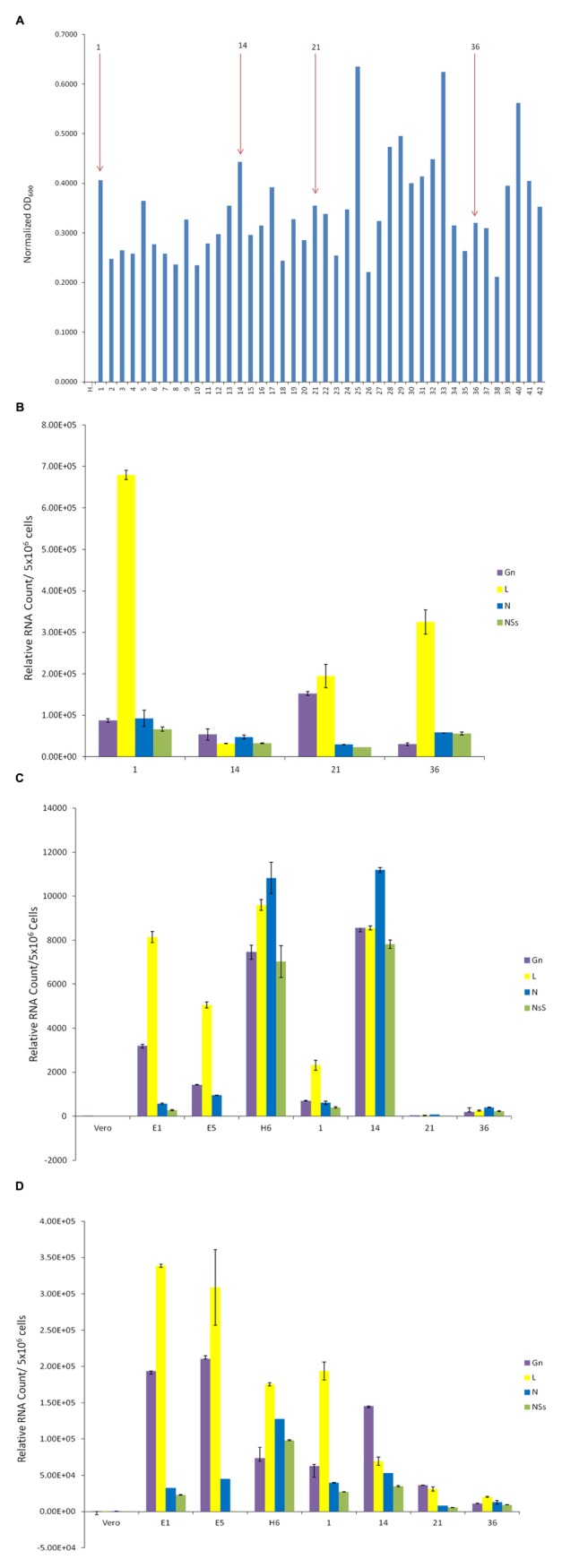
**Presence of RVFV genomic RNA in exosomes.** **(A)** Vero cells were infected with double tagged RVFV for recovery of resistant clones as described in **Figure 1**. Resistant clones were isolated and screened on TLR3 indicator cells. **(B)** qRT-PCR of second generation clones was used to detect presence of RVFV genome in cell lysates. The analysis was performed as described in **Figure 2A**. **(C)** Exosome-enriched preparations were isolated from first and second generation clones via differential ultracentrifugation method. Total RNA was extracted to test for the presence of RVFV genome. **(D)** Exosomes were isolated from first and second generation clones through co-immunoprecipitation of cell supernatants using CD63 Dynabeads. Total RNA was extracted to test for presence of RVFV genome.

We next asked whether we could isolate resistant clones again, this time using another RVFV strain that has been reconstructed to contain epitope-tags in the viral genome. The clone epi-RVFV contains V5-tagged L, and C-terminal Flag-tagged NSs. The virus was synthesized by reverse genetics and was replicated in Vero cells to obtain high titers (generous gift of Drs. Terasaki and Makino, UTMB). Again, similar to experimental design in **Figure 2**, Vero cells were infected with wild type epi-RVFV and resistant clones were isolated and screened using TLR3 indicator cells. Results in **Figure 3A** indicate that resistant clones were able to survive viral infection and subsequently few of the clones were isolated for passages and dilution cloning. Four clones were chosen for the characterization mainly due to ease of isolation and maintenance of clones in cell culture for an extended period of time. We next asked whether these second generation clones contained intracellular viral RNA. Results in **Figure 3B** indicate that all of the second generation resistant clones contained viral RNA, albeit at varying levels. For instance, the genomic RNA in clone #1 contained more L polymerase gene as compared to other clones; clone #21 had the highest level of Gn RNA. These results demonstrate that the resistant clones infected with epitope-tagged RVFV carry genomic RNA similar to the untagged (first generation) resistant clones.

We next isolated exosomes from the second generation clones using differential centrifugation and compared their RNA content with exosomes from the first generation un-tagged clones. Results in **Figure 3C** indicate that indeed the exosome-enriched preparations contained viral RNA in both sets of first and second generation clones. Interestingly, the overall levels of RNA in exosomal preps were less than the intracellular RNAs. Two clones, H6 from first generation and #14 from the second generation, showed the highest level of genomic RNA (all four) in the exosome-enriched preps. Finally, using a well-known exosomal membrane marker (i.e., CD63), we further purified the exosomes from supernatants and characterized for presence of viral RNA. Results in **Figure 3D** indicate that exosomes obtained using CD63-immunoprecipitations (IP with Dynabead – CD63) also contained viral RNA, similar to the differential centrifugation method normally used for exosomal preparation. Interestingly, the overall levels of exosomal CD63-IP viral RNA almost mimicked the exosomal isolation using centrifugation procedure, but contained one log more in the overall RNA contents. Collectively, these results imply that Rift RNA is present not only in intracellular compartment of the resistant clones but also in exosomes.

### Effect of Exosome-Enriched Materials on Recipient Cells

We and others have previously shown that exosomes from latently HIV-1 or HTLV-1 infected cells are capable of controlling survival of recipient uninfected cells (Lenassi et al., 2010; Narayanan et al., 2013; Aqil et al., 2014; Jaworski et al., 2014a). The effects were largely due to either viral proteins or RNA present in their exosomes. Here, we asked whether the exosome-enriched preparations obtained from the Rift resistant clones also were able to control cell survival in recipient cells.

**FIGURE 4:**
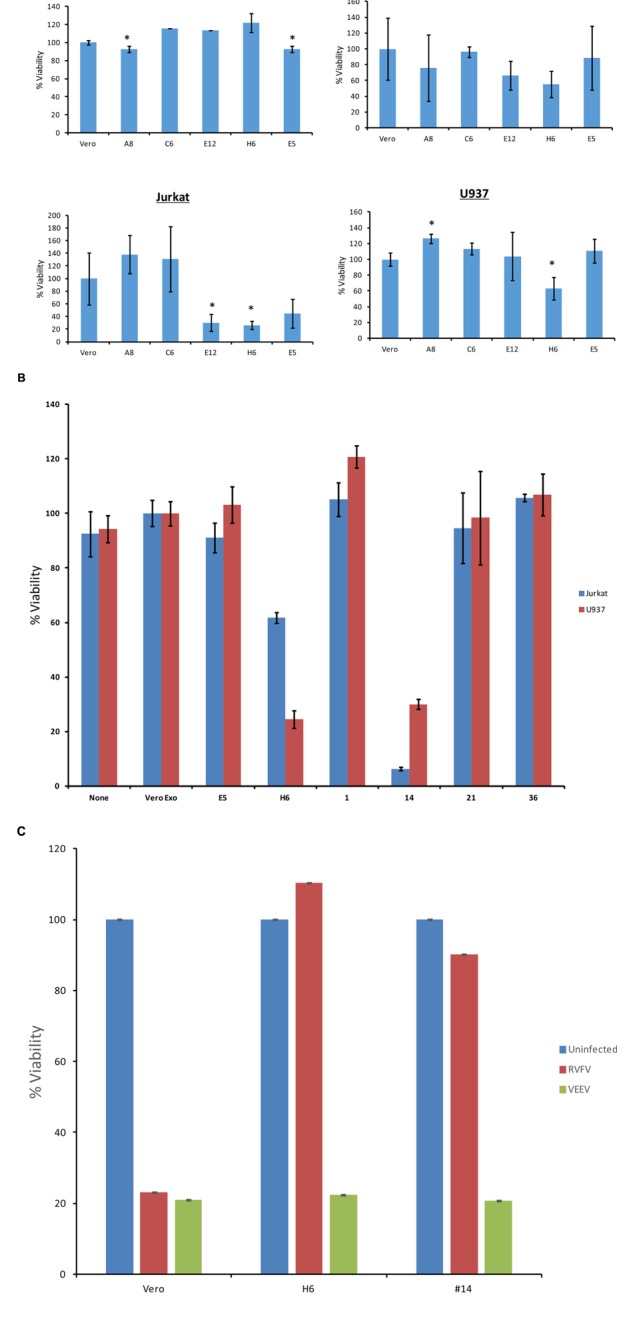
**Effects of exosomes on recipient cells.** **(A)** Approximately 50,000–100,000 cells in 50 μl exosome free media were plated in a 96 well plate. Fifty microliters of supernatant from resistant clones (A8, C6, E5, E12, and H6) and Vero cells were centrifuged 10 min at 14,000 × *g*. Then 50 μl of supernatants were added to cells and allowed to incubate at 37°C for 5 days. Viability was subsequently assayed using CellTiter-Glo assay and values for exosome-free DMEM (control) were used to subtract background. Samples treated with supernatant from uninfected Vero cells were set to 100% and used to normalize the experimental values. The assay was conducted using biological triplicates and error bars indicate ±1 SD. **(B)** Exosomes were isolated through differential ultracentrifugation and 2 μg was added to 50,000–100,000 cells in a 96 well plate. Cells were allowed to incubate at 37°C for 5 days and viability was subsequently assayed using CellTiter-Glo assay. Again, values for exosome-free DMEM (control) were used to subtract background and samples treated with exosomes from uninfected Vero cells were set to 100% and used to normalize the experimental values. The assay was conducted using biological triplicates and error bars indicate ±1 SD. **(C)** Approximately 100,000 mid-log Vero cells, H6 or clone #14 were treated with either RVFV or VEEV (TC83) at MOI = 1 and incubated at 37°C for 5 days. Samples were processed for cell viability using CellTiter-Glo in a microtiter plate. Triplicate samples were treated with either of the two viruses. ^∗^*p* < 0.05.

We designed two sets of experiments on indicator cells using either supernatants or concentrated exosomes. Approximately 50–100,000 cells were plated in a 96 well plate and supernatants from resistant clones (A8, C6, E1, E5, E6, E12, and H6) and Vero control cells were added to cells and allowed to incubate at 37°C for 5 days. Viability was subsequently assayed using CellTiter-Glo. Results in **Figure 4A** indicate that culture supernatants from various first generation clones had no effect on Vero cells after 5 days of culture, perhaps due to the fact that initial clones were obtained from Vero cells. These exosome-containing materials were then added to lymphocytes or monocytes for 5 days. No additional culture supernatant or media was added during the experiment. Results in **Figure 4A** indicate that viability of all three cell types was altered with the exosome-containing culture supernatants s from certain clones, including E12, H6 and E5. Interestingly clone H6 showed the most consistent result among all three indicator cells.

We next enriched exosomes through differential centrifugation from both selected first and second generation clones, treated target cells and performed cell viability assay on both lymphocytes and monocytes. Results in **Figure 4B** indicate that clones H6 and #14 were effectively inhibiting cell viability in both cell types following 5 day-incubation. Interestingly, clones H6 and #14 contain high levels of all four RNAs from RVFV. Other clones including #1, 21, and 36 had lower levels of all RNAs with minimal cell death. Clone E1 had lower N and NSs levels with minimal death and clone E5 was missing Gn altogether with no apparent cell death. Finally, it is important to note that all of these cells were sensitive to infections with other RNA viruses including VEEV. For this we performed another set of experiments where we treated both clone H6 and #14 with RVFV and VEEV and kept the cultures at 37°C for 5 days. Results in **Figure 4C** indicate that both clones were resistant to RVFV infection but sensitive to VEEV infection. Positive control Vero cells were sensitive to both RVFV and VEEV infection, indicating that resistant clones were still sensitive to other RNA viral infections but not to the original parent virus. Overall, these results suggest that the death of recipient cells may be related to high level of viral RNA in exosomes.

### Presence of Viral Proteins in Exosome-Enriched Preparations

**FIGURE 5:**
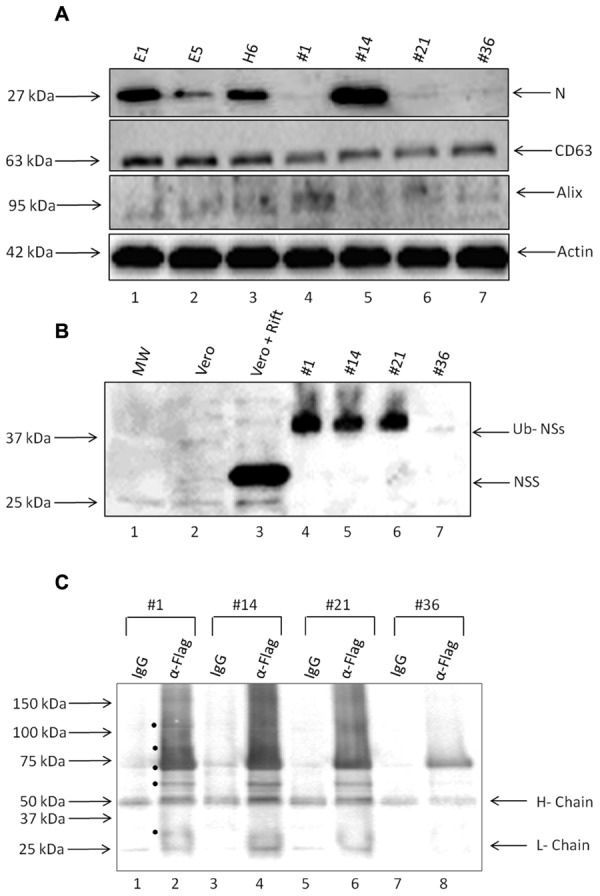
**Presence of viral proteins in exosomes.** **(A)** Enriched exosomes were isolated through differential centrifugation and were analyzed by Western Blotting with antibodies to N protein (generous gift from Dr. Connie Schmaljohn; 1:500), CD63 (ab8219 1:500), Alix (sc-49268 1:150), and Actin (ab49900 1:5000). **(B)** Exosome-containing preparations obtained from resistant clones and corresponding WCEs were analyzed by Western Blots with antibodies to NSs Flag (sc-807 1:200) and Ubiquitin (1:500). **(C)** Three hundred and fifty microgram of purified exosomes was used for lysis, using Freeze-Thaw and TNE 50 + 0.1% NP-40 and immunoprecipitation using either IgG or anti-flag antibody (10 μg each). Samples were IPed overnight; protein A + G beads added (30% slurry) the next day for 2 h, centrifuged and washed. Bound samples were run on a gel for western blot using anti-ubiquitin antibody (1:500). Black circles indicate potential Ubiquitinated NSs proteins present in the exosomes.

Presence of Rift viral RNA promoted us to ask whether viral proteins were also present in these exosome preparations. We performed western blots against two critical viral proteins: N and NSs. Antibody against N protein was readily available (generous gift of Dr. Connie Schmaljohn, USAMRIID) and we also utilized anti-FLAG- antibody to detect NSs protein. Results in **Figure 5A** indicate that clones E1, E5, H6, and #14 contained varying amounts of N protein in the exosome-enriched materials. The presence of N protein was independent of N RNA, as apparent in clones #1, 21, and 36. Other exosomal markers including CD63, Alix, and actin were expectedly present in all tested clones.

Finally, we asked whether NSs protein was also present in these preparations. We used only second generation clones using epi-tag antibody for these experiments which allowed probing for the FLAG tag as an indication of the presence of NSs. Results in **Figure 5B** indicate that NSs was present in three out of four preparations; however, the intracellular NSs (from Rift virus infected cells) were mostly unmodified whereas the NSs protein in the exosome-enriched preparations showed a protein of a slightly higher molecular weight. We recently have obtained data from both HIV-1 and HTLV-1 exosomes indicating that many critical viral proteins may be modified including ubiquitination (unpublished results). Therefore, we performed a series of immunoprecipitation (IPs) from our exosomes using anti-flag antibody directed toward NSs, followed by western blot using anti-ubiquitin antibody. Our rationale for these experiments, as stated above, were that NSs might be ubiquitinated in these exosomes as we find most proteins expressed and packaged into exosomes potentially modified including ubiquitination (data not shown). Results in **Figure 5C** indicate that there are various forms of the modified NSs in the exosomes when using anti-flag antibody. Although IgG control immunoprecipitation did bring down some non-specific proteins, there were more bands in the anti-flag IPs from the four exosome clones used in the assays. Interestingly, there were less of the ubiquitin-NSs in clone #36 as compared to others. Collectively, these data imply that certain exosomes contain not only viral RNA but also varying levels of viral N and modified NSs proteins.

### Effect of Exosome on the Recipient Cell Apoptosis Machinery

**FIGURE 6:**
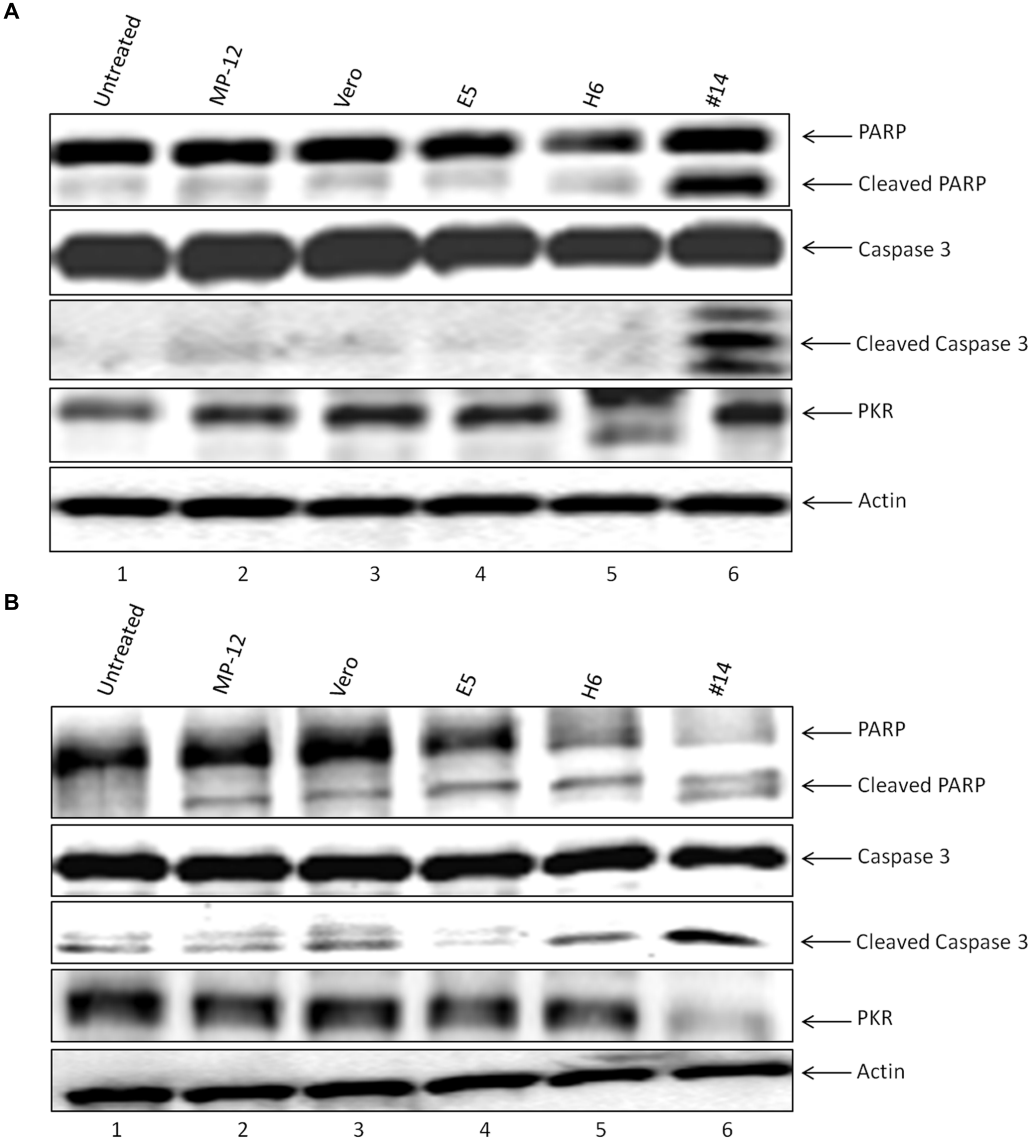
**Effect of exosomes on the recipient cell apoptosis machinery.** **(A)** Jurkat cells treated with exosome-enriched preparations from resistant clones were analyzed by Western blotting with antibodies to markers of apoptosis, which included Caspase 3 (sc-7148 1:200), PARP (sc-7150 1:200), and PKR (sc-707 1:200), or Actin (ab49900 1:5000). **(B)** U937 cells were treated similar to **(A)** and extracts were used for western blot analysis probing for the same apoptosis markers.

We next asked whether classical markers of apoptosis were present in the recipient cell extracts treated with exosome-enriched populations. We performed western blots for markers PARP, caspase 3, as well as PKR degradation, a known marker of NSs target in cells (Habjan et al., 2009). We treated both T-cells (**Figure 6A**) and monocytes (**Figure 6B**) for 5 days and subsequently obtained whole cell extracts for western blot analysis. We used either Rift infection (MOI = 0.1) or uninfected Vero exosomes as positive and negative controls. Our rationale for these experiments were based on the fact that Cell Titer Glo assay alone (data in **Figure 4**) does not differentiate between various stages of cell viability. Data in **Figure 4A** indicate that PARP and Caspase 3 cleavage was mostly seen in recipient T-cells treated with exosome clone #14. This was also somewhat consistent in monocytes treated with clone #14 exosomes (**Figure 4B**). Interestingly, clone H6 treated cells didn’t show appreciable levels of PARP or Caspase 3 cleavage in either of the cell types, indicating that the mechanism of action for these two clones may be very different in treated recipient cells. Finally, for treatment with clone #14, the degradation of PKR was much more pronounced in monocytes as compared to T-cells, implying that the NSs activity may be higher in these cells. Collectively, these results imply that exosome treated recipient cells may undergo a generalized apoptosis.

### Effect of Resistant Clone Supernatants on Vero and Immune Cells

**FIGURE 7:**
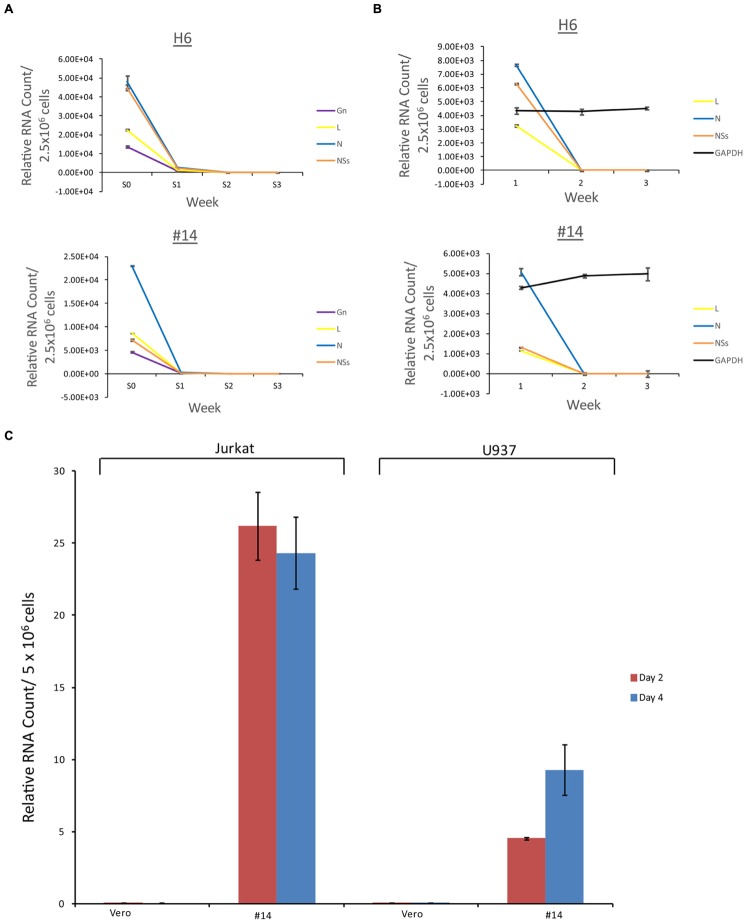
**Effect of resistant clone supernatants on Vero cells.** **(A)** Supernatants (1 ml) from clones H6 and #14 were added to Vero cells (2.5 × 10^6^/ml in complete media in a 24 well plate; ∼35% confluency) overnight, washed 24 h later and supplemented with complete media. Each week, supernatants (1 ml) were collected and concentrated using mixture of nanoparticles NT080 + NT082 (to concentrate exosomes; 50 μl of 30% slurry) and NT086 (to concentrate potential virus; 50 μl of 30% slurry) overnight at 4°C. Samples were pelleted the next day, washed, and total RNA was isolated for qRT/PCR. “S0” denotes the starting material and “S1–3” was supernatants from treated Vero cells in weeks 1–3. **(B)** Similar to **(A)**, except that the total RNA was isolated from the cell pellets prior to qRT/PCR for Rift RNAs. GAPDH served as internal control for RNA expression. **(C)** Total RNA was isolated from Jurkat and U937 cells treated with exosome-containing preparations from Vero or resistant clone #14 after 2 and 4 days. qRT-PCR analysis were conducted with primers specific for NSs.

We next asked whether the apoptosis observed in the immune cells (**Figure 4**) were the result of potential presence of mutant viruses contaminating our resistant clone preparations. This is critical since most of the exosomes obtained and characterized to date (clones H6 and #14) do contain characteristics of a potential virus (i.e., mutant) in the supernatants. We therefore designed experiments where we added unprocessed supernatants from the resistant clones to normal Vero cells (susceptible to Rift infection) and carried these cells for 3 weeks. Unfiltered supernatants from both clones H6 and #14 were then added to fresh Vero cells and washed 24 h later. Fresh media was added and samples (both supernatants and cells) were collected at the end of weeks 1, 2, and 3. The cells at the end of week 1 and 2 were split 1:3 to avoid over growth in plates. Independent supernatants from week 0 (starting material) and weeks1, 2, and 3 were treated with nanoparticles NT080, NT082 (to concentrate exosomes) and NT086 (to concentrate potential virus) prior to qPCR for presence of Rift genome. We have previously shown that dilute viral or exosomal samples can be concentrated and enriched using specific nanoparticles (Jaworski et al., 2014b). Data in **Figure 7A** shows that Rift RNA was present in the resistant clone supernatants (starting material; S0) and almost completely absent in supernatants from treated Vero cells in weeks 1–3 (S1–3). We also looked at the intracellular RNA level from these treated cells and found that the Rift RNA was only present in week 1 samples and completely absent in weeks 2 and 3 samples (**Figure 7B**). Cells at weeks 1–3 were actively growing as evident by duplication and presence of GAPDH. We next asked whether exosomes from clone #14, that were involved in the induction of apoptosis in immune recipient cells, had the capacity to replicate their genomic RNA in these cells. Our rationale for these experiments is based on data demonstrating that exosomes from HCV infected cells contain viral genomic RNA that can enter into uninfected cells and replicate independent of its specific receptor (Masciopinto et al., 2004; Bukong et al., 2014). This would be in comparison to an active viral replication, where viral RNA replicates to ∼10^6^ copies in 24–48 h after infection. Therefore, we treated both Jurkat and U937 cells with clone #14 or control exosomes from uninfected Vero and isolated total RNA at 2 and 4 days followed by qRT-PCR for presence of viral genomic RNA. Results in **Figure 7C** indicate that the Rift RNAs did not replicate in either cell type, further implying that components of replication machinery may be missing in these exosome-enriched samples, hence resulting in an eventual loss of genomic RNA in the recipient cells. Collectively, these data further imply that the resistant clones do not contain mutant viruses that could potentially replicate with slower kinetics in susceptible cells.

### Effect of RVFV on Immune Cells

**FIGURE 8:**
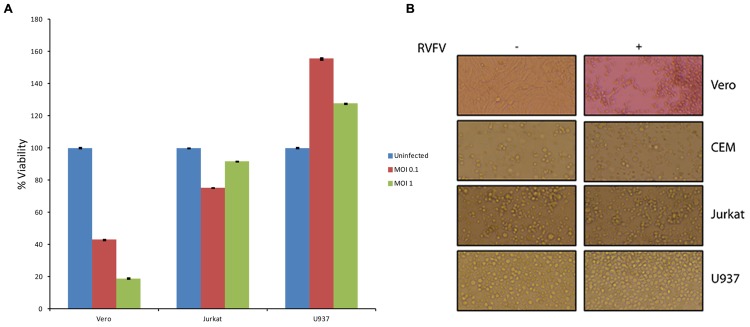
**Effect of RVFV infection in immune cells.** **(A)** Immune cells including T-cells (Jurkat) and monocytes (U937) were grown to log phase of growth in complete media (1.5 × 10^6^ cells/ml). RVFV (epitope-tagged MP12) at MOI of 0.1 and 1.0 were used for infection of immune cells and Vero cells as a positive control. Cultures were incubated for 5 days as 37°C (without removing the virus) and cell viability were assayed using CellTiter-Glo. Results in **(A)** are from three independent experiments. Similar results were also observed with the wild type un-tagged MP12 (data not shown). **(B)** Similar to **(A)** except cells infected at MOI of 1 after 5 days were used for cell morphology using light microscopy.

One of the fundamental questions regarding our work on exosomes is whether exosomes released from infected cells could play a critical role in the pathology of the disease in comparison to the viral release. This is an important question, since viral infection usually results in release of high titer viruses over a period of time which may overwhelm and mask any potential exosomal effect on the recipient cells. Along these lines, data in **Figures 4** and **7** indicate that exosomes can regulate the recipient immune cells and cause apoptosis over a period of time. However, it was not clear to us whether RVFV could also cause apoptosis of immune cells *in vitro*. To answer this, we performed an experiment where T-cells (Jurkat) and monocytes (U937) were treated with two concentrations of RVFV (MOI = 0.1 and 1.0) and incubated with the virus for 5 days. In our initial set of experiments, we monitored these cells everyday by microscopy and trypan blue staining (data not shown). Subsequently quantitative experiments were performed in microtiter plate and assayed for viability using CellTiter-Glo assay. Interestingly, we observed complete resistance of the immune cells to apoptosis by RVFV over an extended period of time (**Figure 8A**). As expected, positive control Vero cells were apoptosed following infection. This is also reflected in the cell morphology as shown in **Figure 8B** where Vero cell monolayers were rounded up following RVFV infection but no apparent cellular changes were observed in T-cell and monocytes. Collectively, these results imply that immune cells may be resistant to RVFV-induced apoptosis, whereas exosomes could potentially target immune cells for destruction. This dual action of viral infection of specific target cells and apoptosis of immune cells from exosomes could contribute to the overall pathology of RVFV infection *in vivo*.

## Discussion

In recent years, exosomes have emerged as essential and critical components of intercellular communication during viral infection related to disease states, including cancers and spreading of the viral infections. We have previously shown that exosomes from virally infected latent cells have the ability to control gene expression and cell viability in the recipient cells (Narayanan et al., 2013; Jaworski et al., 2014a). Here, we tested whether Rift infected cells could also secrete exosomes that may be able to control the fate of the recipient cells. We utilized a scheme to generate reliable clones that survived RVFV infection and that their exosomes contained potential viral RNA and/or proteins. Using *in vitro* infection of Vero cells, we were able to generate multiple resistant clones that could be passaged up to at least 50 times and still retained both viral RNA and proteins in the exosome-enriched preparations. We performed these experiments in two independent sets of infections: with wild type MP12 and with an epitope-tagged MP12. Using TLR3 as an initial read out assay, we obtained clones that produced exosome-containing secretions in 5 day-cultures that contained varying levels of genomic RNA and viral proteins. These clones could be grown to large scale, which simplifies purification schemes for downstream functional assays. Interestingly, these clones were all resistant to Rift infection, but not to other viruses including VEEV, indicating that the mechanism of resistance may be unique and not general for most incoming RNA viruses. These data also indicate that the Rift replication/transcription machinery must still be somewhat intact in these resistant clones, as both RNA and proteins were present after continuous passage and replication. It is therefore tempting to speculate that the viral assembly in these cells may be blocked, and/or that there is a competition between viral or exosomal release as both entities presumably utilize the same exit machinery. The broader implications are that if a cell is deficient in one of the many components of the ESCRT (release) pathway, or cells which contain mutant viral genome for release, or cells that are under drug selection pressure, they might collectively favor exosomal release containing viral components over viral progeny formation and release. Future experiments using the CRISPR system as well as mutant viruses in various ORFs could potentially better define these overlapping mechanisms.

Previous work with HCV (Masciopinto et al., 2004) demonstrated the presence of viral RNA in exosomes released from infected cells. These exosomes spread viral infection to the neighboring cells, indicating that the genomic RNA was capable of replicating in cells. We asked whether the same findings may be true for the RVFV resistant clones. Consistent with our previous work from HIV-1 and HTLV-1, we were unable to observe Rift replication in the recipient cells, indicating that the full replication machinery is not transferred in these exosome-containing secretions. Therefore, HCV RNA transfer in exosomes may have a different packaging set of signals compared to other nuclear or cytoplasmic viral RNAs, which may cause it to transfer complete replication machinery through exosomes. We also performed a time course study where supernatants from resistant clones were added back to uninfected Vero cells. These cells survived replication for the duration of the study (3 weeks) and did not produce wild type viral progeny. Importantly supernatants from these newly treated cells did not show presence of viral RNA or virus in the supernatants. We did, however, observe cell associated viral RNA (10-fold less) in samples from week 1, which may be due to presence of the input exosomal RNA or a very slow growing mutant virus population. Either way, the effect on host Vero cells were not deemed significant compared to the results from immune Jurkat and U937 cells which showed apoptosis when treated for few days.

Using HIV-1 as our model system for presence of viral RNA in exosomes (Narayanan et al., 2013), we have recently observed binding and activation of number of TLRs to these RNAs when using the exosomes from latently infected cells (Sampey et al., 2015). Here, we used a functional TLR3 assay as a generic readout for TLR and NF-κB activation. We consistently have observed activation of TLR3 in these assays when using exosomes obtained from resistant clones. This further suggests that the exosomal cargo may have functional properties in the recipient cells, including activation of signal transduction for genes involved in cell fate.

Our individual clones also demonstrate a role in apoptosis of recipient cells that may depend on the expression of viral RNA and proteins. For instance, clone H6 shows appreciable amounts of all RNAs and some N protein whereas clone #14 contained viral RNAs as well as increased N protein levels and NSs. We currently cannot rule out whether NSs protein is present in clone H6 due to lack of antibody; however, the overall effect may result in delayed apoptosis compared to clone #14 with higher levels of detectable NSs. It is important to note that treatment with exosomes from clone #14 exhibits all the classical signs of Rift infection in recipient cells, including presence of apoptosis markers and PKR cleavage. Although we cannot rule out the presence of other viral proteins in clone #14, presence of all viral RNAs, N, and NSs protein may be responsible for apoptosis in both T-cells as well as in monocytes.

Our data using resistant clones clearly suggest that exosomes from infected cells may contain viral RNA and proteins. The net effect of these exosomes may be multidimensional, but at least some may be capable of inducing apoptosis in uninfected cells. We speculate that Rift infection *in vivo* may also allow formation of exosomes that persist for an extended period of time. Whether those exosomes contribute to the overall pathology of animals or humans is not clear; however, they could potentially play a role in compromising the immune system which would then allow for better replication of the virus. In essence, these vesicles could act as a “decoy” to dampen the innate (or potentially acquired) immune response in hosts for a rapid viral spread. Alternatively, they could contribute to death of not only adult immune cells but also pluripotent stem cells, resulting in the spontaneous abortion observed in animals. Our unpublished data using either virus or exosomes on human stem cells point to this direction. Future experiments using animal models and inhibitors that allow blocking of exosome formation from infected cells may uncover their true potential in pathogenesis *in vivo*.

## Conflict of Interest Statement

The authors declare that the research was conducted in the absence of any commercial or financial relationships that could be construed as a potential conflict of interest.
